# The diverse life-course cohort (DLCC): protocol of a large-scale prospective study in China

**DOI:** 10.1007/s10654-022-00894-1

**Published:** 2022-07-19

**Authors:** Huijing He, Li Pan, Yaoda Hu, Ji Tu, Ling Zhang, Minying Zhang, Gongshu Liu, Juxiang Yuan, Qiong Ou, Zhiwei Sun, Jing Nai, Ze Cui, Jingbo Zhang, Jing Wang, Jianhui Wu, Xiaoyan Han, Yujie Niu, Xiaoming Li, Dongqing Hou, Chengdong Yu, Chenchen Jiang, Qihang Liu, Binbin Lin, Guangliang Shan

**Affiliations:** 1 Department of Epidemiology and Statistics, Institute of Basic Medical Sciences, School of Basic Medicine, Chinese Academy of Medical Sciences, Peking Union Medical College, Beijing, China; 2 Department of Epidemiology and Health Statistics, School of Public Health, Capital Medical University, Beijing, China; 3 School of Medicine, Nankai University, Tianjin, China; 4 Tianjin Women’s and Children’s Health Center, Tianjin, China; 5 School of Public Health, North China University of Science and Technology, Tangshan, Hebei China; 6 Hebei Province Key Laboratory of Occupational Health and Safety for Coal Industry, North China University of Science and Technology, Tangshan, Hebei China; 7 Sleep Center, Department of Pulmonary and Critical Care Medicine, Guangdong Provincial People’s Hospital, Guangdong Academy of Medical Sciences, Guangdong Provincial Geriatrics Institute, Guangzhou, China; 8 Department of Preventive Medicine, School of Public Health, Hebei University, Baoding, Hebei China; 9 Clinical Laboratory, Bejing Hepingli Hospital, Beijing, China; 10 Hebei Provicel Center for Diseases Prevention and Control, Shijiazhuang, Hebei China; 11 Beijing Physical Examination Center, Beijing, China; 12 Chaoyang District Center for Disease Control and Prevention, Beijing, China; 13 Hebei Key Laboratory of Environment and Human Health, Shijiazhuang, China; 14 Department of Occupational Health and Environmental Health, Hebei Medical University, Shijiazhuang, China; 15 Department of Epidemiology, Capital Institute of Pediatrics, Beijing, China; 16 Child Health Big Data Research Center, Capital Institute of Pediatrics, Beijing, China

**Keywords:** Cohort study, Noncommunicable chronic diseases, Environmental health, Biobank, Design

## Abstract

The Diverse Life-Course Cohort (DLCC) is a large-scale prospective study including around 130,000 participants in mainland China. The primary aims of DLCC include contributing to knowledge on noncommunicable chronic disease determinants, particularly cardiometabolic diseases, and exploring the long-term effect of ambient air pollutants or other environmental risk factors on health among all-age populations. The cohort consists of several sub-populations that cover the whole life-course and diverse resources: from premarital to adolescents, adults from workplace and communities ranged from 18 to 93 years old. Baseline assessment (2017–2021) included face-to-face standardized questionnaire interview and measurements to assess social and biological factors of health. Blood samples were collected from each participant (except for children younger than 6) to establish the biobank. DLCC consists of two visits. Visit 1 was conducted from 2017, and 114850 individuals from one of the world-class urban agglomerations: Beijing, Tianjin, and Hebei area were recruited. By the end of 2021, at least one follow-up was carried out, with an overall follow-up rate of 92.33%. In 2021, we initiated Visit 2, newly recruited 9,866 adults from Guangdong province (South China) and Hebei province (Central China), with research focuses on the comparations on ambient pollution hazards and other unique dietary or environmental risks for health. The baseline survey of Visit 2 was finished in July 2021. DLCC is still ongoing with a long-term follow-up design, and not limited by the current funding period. With reliable data and the well-established biobank which consists of over 120,000 individuals’ blood samples, DLCC will provide invaluable resources for scientific research.

## Introduction

The Diverse Life-Course Cohort (DLCC) is a population-based prospective cohort study initiated from 2017, covering the whole life course from prenatal life to aged people in China. DLCC was conducted by the Institute of Basic Medical Sciences (IBMS), Chinese Academy of Medical Sciences (CAMS).

The rapid growth of cardiovascular diseases (CVDs) and other noncommunicable chronic diseases (NCDs) in China in the past decades necessitated systematic investigations on etiology and target prevention for reduction of disease burden [1]. Given that most NCDs have a prolonged subclinical phase, large-scale and long-term longitudinal studies are the best tools to disentangle the complicated role of etiological factors that interact over time[2]. In response to a raising need to better understand life conditions, in 2016, supported by the Ministry of Science and Technology, a million-level cohort was scheduled to cover diverse populations in mainland China. The Beijing-Tianjin-Hebei (BTH) general population cohort is one of them.

The BTH region is one of the world-class urban agglomerations in China. As the political center and third largest economy in China, the BTH region accounts for 8.1% of China’s population. This area is experiencing rapid socio-economic development and urbanization, and consequently caused severe air pollution and huge changes in health-related behaviors [3]. As one of the most air-polluted areas in China, the concentration of PM_2.5_ has increased from 2013 to 2016 [4, 5]. The health hazard of ambient pollutants has therefore drawn the attention of public health and scientific research. In 2017, the first visit (Visit 1) was initiate. By the end of 2021, participants in Visit 1 have experienced at least once follow-up and an overall 114,850 individuals have been involved.

In 2021, the second visit (Visit 2) was initiated, with another two areas, Shantou and Meizhou cities (including three different kinds of culture: “Chaoshan”, “Hakka” and island culture in South China) in Guangdong Province (5655 participants recruited in the baseline) and Baoding city in Hebei Province (4211 participants in the baseline). The two newly enrolled areas have some unique characteristics in dietary patterns, significant difference in environmental risk factors (such as concentration of ambient air pollutants), and NCDs prevalence. With low ambient pollutant exposure level, Shantou and Meizhou cities enable us to conduct comparative studies on different ambient pollution patterns on multiple health outcomes, and additionally allows for the exploration of health hazard of long-term exposure to low level ambient pollutants. The newly enrolled study sites, Nan-Ao island in Shantou city, which is selected as the representation of island culture, is a relatively isolated place with native residents living with unchanged customs. It provides the ideal population to study the environmental risk factors as well as the interaction with genetic backgrounds for health, thus allows migrant epidemiology research as people may float to developed urban areas due to more job opportunities. Therefore, in Visit 2, the health hazard of ambient pollutants in varied concentration pattern, together with other environmental risk factors, especially dietary determinants, on health was the research focus.

**Fig. 1:**
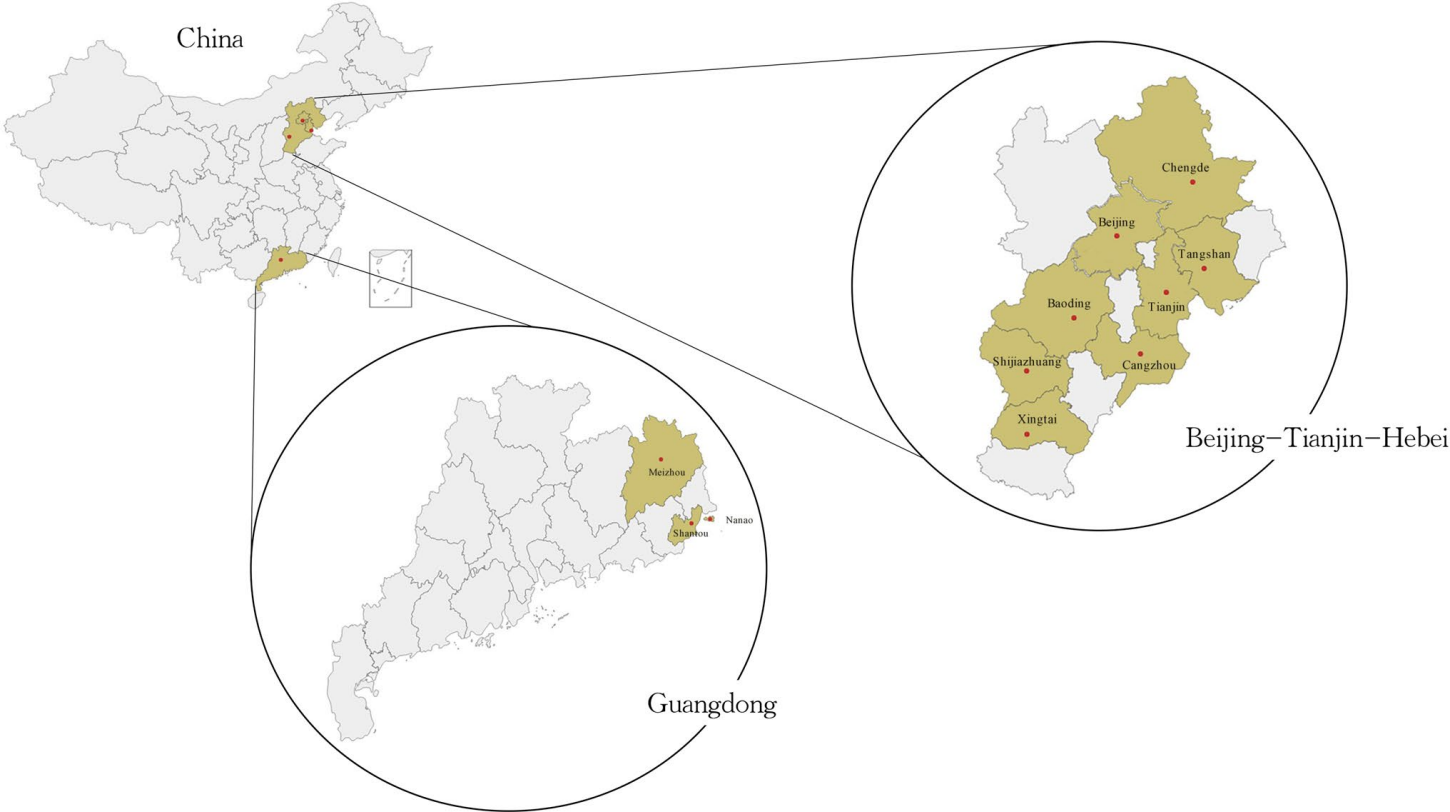
The location of study sites in DLCC

The location of DLCC was shown in Fig. 1. 124,716 participants aged from 0 to 93 have been recruited in the baseline survey.

The primary goals of DLCC are: (1) to describe the health variations across different populations in the whole life course in disease susceptibility, health phenotypes, and natural history of specific noncommunicable diseases (NCDs) after long-term follow-up; (2) to identify environmental and genetic determinants for cardiometabolic disease (CVD) and other NCDs, from early life to senior age and (3) to examine the health hazards of ambient air pollutants on health in different geographic areas.

DLCC is one of the largest prospective cohorts established in China thus far and is an invaluable resource for health research and clinical study. In addition, by storing blood samples of more than 120,000 individuals with high population heterogeneity, it will allow reliable assessment of genetic and other factors for diverse health outcomes. We believe that the rich dataset and biobank established by DLCC will contribute to deeper understanding of NCDs etiology, and to the development of optimal strategies for health care for all-age general population. In the future, novel, state of the art methods including genome-wide association studies, metabonomic and proteomic methods will be used to understand the disease pathogenesis and progress.

## Study design and population

**Fig. 2:**
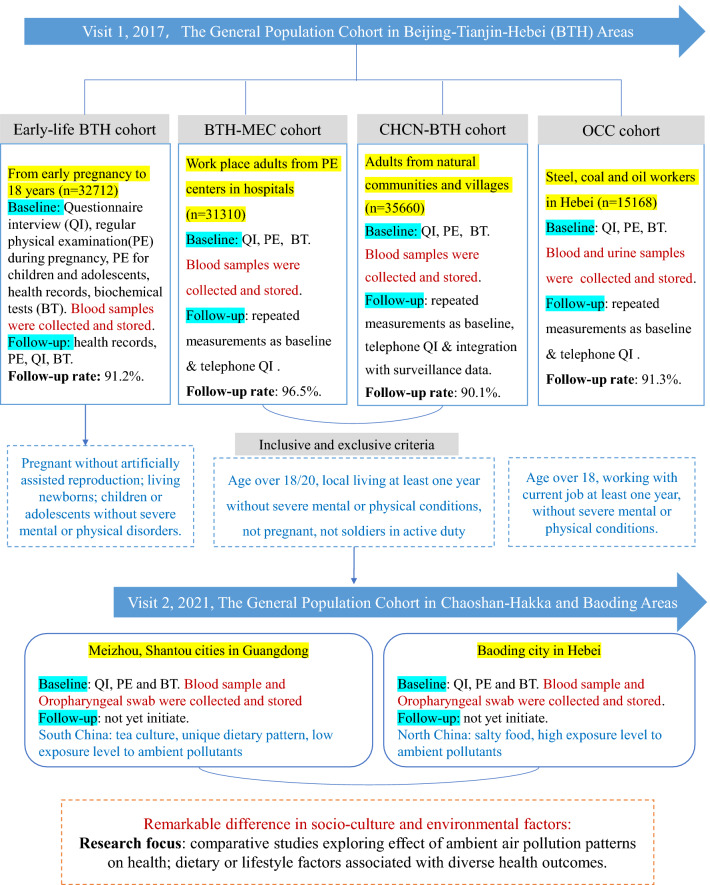
Cohort design and measurements of DLCC

The overall study design is presented in Fig. 2. DLCC was composed by several sub-cohorts covered subjects from early life to senior age, with diverse population characteristics:

1. The cohort from early pregnancy to adolescents in BTH area (Early-life BTH cohort), which enrolled an overall 32,712 subjects in the baseline. Pregnant women at early stage were selected from three-tier antenatal healthcare system in Tianjin, including community-level primary healthcare center, district-level Women and Children’s Health Centers (WCHC) and other tertiary hospitals, and city-level Tianjin WCHC and other tertiary hospitals [6]. Finally, 5920 pregnant women participate the study. Multi-stage stratified sampling method was used to select children and adolescents. Kindergartens, elementary and high schools were firstly selected from different districts with difference in the urbanization and economic development level in the BTH areas, then classes in the first grade of kindergartens and different grades in elementary and high schools were selected. Children and adolescents in the selected classes were all invited to participate in the survey. Finally, 3952 children aged 3–5 years and 22,840 children aged 6–18 years participate the study. By covering population at the early stage of lifespan, this sub-cohort aims to understand the influence of early life risk factors of adulthood NCDs and key determinants for reproductive and youth’s health.
2. The BTH Medical Examination Cohort (BTH-MEC), which enrolled 31,310 adults aged 18 and above from physical examination centers in hospitals in BTH areas. To select participants, we first randomly selected two or three medical examination centers by systematic sampling among records of more than 200 physical examination centers in tertiary care hospitals in the BTH area, then stable employees in organizations, institutions, and companies were selected with multiple labor categories (white collar, pink collar, or blue collar) from each selected physical examination center. By collecting data from regular physical examinations, this sub-cohort focuses on clinical health profiles and exploration of new biomarkers of NCDs, and effect of special workplace exposures on health outcomes. Importantly, recruiting participants from physical examination hospitals helps to alleviate the imbalance of sex and age proportion (most were retired female participants), which always occurs in epidemiological field work when recruiting people from communities.
3. The cohort on chronic disease of community natural population in BTH region (CHCN-BTH cohort), which enrolled 35,660 adults at baseline aged 18 and above from urban communities and rural villages[7]. This sub-cohort focuses on aging and its related health risks among senior population. Multi-stage stratified cluster sampling method were performed to recruit participants: firstly, we chose districts (in Beijing and Tianjin, both are municipalities in China, equal to provincial level) or cities (in Hebei Province), then streets in the districts of Beijing and Tianjin, districts or counties in the cities of Hebei were selected; in the third stage, communities were chosen from urban streets or districts, villages were chosen from counties. In the final stage, residents living in the selected communities and villages were all invited to participate in the baseline survey.
4. The occupational cohort (OCC cohort) includes 15,168 steer workers, coal workers, and oil workers. This sub-cohort was conducted in Hebei province, where most heavy industry factories located. Cluster sampling method was used to recruit participants. 7628 steel workers, 4440 coal workers and 3100 oil workers were recruited, respectively. Data on occupational risk factors, such as rotating working pattern, special toxic exposures at workplace were collected to explore their association with multiple health outcomes.

The above four sub-cohorts formed Visit 1 of DLCC. By including participants from prenatal to senior age, the cohort provides comprehensive perspectives for health estimation. Workplace exposures collected in the BTH-MEC and OCC cohorts will additionally contribute to establishing healthier working environments.

(5) The Chaoshan-Hakka-Baoding-general population cohort (CHB cohort) which included 9,866 participants aged 20 and above from urban communities and rural villages from Baoding city of Hebei Province, Shantou and Meizhou cities in Guangdong Province. A multistage stratified sampling method, same with the sampling method used in the CHCN-BTH cohort, was used to select subjects. Dietary and gout-specific information was additionally collected in this cohort. The CHB cohort formed Visit 2 of DLCC, of which comparative studies on environmental risk factors, health hazard of ambient pollutants in varied geographic areas representing different Chinse cultures will be given more concern.

The inclusion and exclusion criteria are available in Fig. 2. Generally, individuals that had lived in the project area for at least one year at the time of the study, aged over 18 (for adults’ cohorts), without severe mental or physical condition, not soldiers in active duty were recruited. People recruited from communities and villages had the same inclusive and exclusive criteria with our previously conducted China National Health Survey (CNHS)[8].

The (1)–(4) sub-cohorts were all initiated in 2017 (Visit 1), and at least one follow-up has been conducted in each. The fifth sub-cohort was conducted in 2021 (Visit 2), which led by the same faculty who participated in the previous cohort from CAMS. E-cigarette smoking, dietary pattern, tea consumption, sleep conditions, HPV infection in oropharynx, oropharyngeal health and gout-specific information was additionally collected. The basic characteristics of DLCC were summarized in Table 1.

**Table 1:** Phenotypic data collected in DLCC

Data collection strategy	Measurements
Questionnaire interview	Basic characteristics: sex, birthday, educational attainment, occupation, marital status, urban or rural residence, personal income, medical insurance Life-style risk factors: cigarette smoking, alcohol intake, physical exercise, sedentary behavior, dietary information, sleeping condition, mental health assessment (depression and anxiety) Personal medical history: hypertension, diabetes, dyslipidemia, hyperuricemia, gout, cardiovascular disease, stroke, cancer, fracture, infectious diseases (hepatitis and tuberculosis). etc. Medication use Family disease history: hypertension, diabetes, dyslipidemia, hyperuricemia, gout, cardiovascular disease, stroke, cancer, etc Reproductive factors: menstruation and menopausal information for women, history of gynecological surgery
Anthropometry and Physical examination	Height, weight, waist, hip circumference, body composition (body mass index, body fat percentage, fat mass, fat free mass, muscle mass, vertical fat index) Systolic and diastolic blood pressure Hand grip strength (predominant hand) Electrocardiogram: heart rate, PR interval, QRS duration, QT interval, etc Bone mineral density test Respiratory function test: tidal volume (VT), minute ventilation volume (MV), vital capacity (VC), forced expiratory volume in one second (FEV1), forced vital capacity (FVC), etc Oropharyngeal examination Ultrasound measurement
Biochemical tests	Fasting glucose, serum lipids (total cholesterol (TC), triglyceride (TG), high density lipoprotein cholesterol (HDL-C), low density lipoprotein cholesterol (LDL-C), lipoprotein (a)), hemoglobin A1c, serum uric acid, alanine transaminase (ALT), aspartate aminotransferase (AST), γ-Glutamyl transpeptidase, urea, creatinine, etc
Complete blood count	Red blood cell count, white blood cell count, hemoglobin, platelet, hematocrit increases (HCT), mean corpuscular volume (MCV), neutrophil count, lymphocyte count, monocyte number, eosinophil count, basophil count, etc

The integration of the sub-cohorts were based on the following considerations: (1) inclusion of populations covering the whole life course, from prenatal to senior age, which provided unique resource to understand disease pathogenesis and risk factors in the whole life course; (2) inclusion of populations with different socioeconomic levels, varied environmental, workplace and occupational exposures; (3) recruitment of populations with low migration rates that providing an advantage for long-term follow-up; (4) led by the same faculty under consistent quality control measurements and shared common philosophy and values (for promotion of wellbeing of the whole society, but not only scientific research purpose).

### Data collection and baseline examination

DLCC collected data on demographic and socioeconomic information, health related lifestyle factors, anthropometric measures, laboratory tests and clinical profiles. An overview of data collection is shown in Table 2.

**Table 2:** Baseline characteristics of the participants recruited in DLCC

Characteristics	Male		Female		Overall	
	*n*	%	*n*	%	*n*	%
*Children and adolescents*						
0–2	3137	18.77	2783	17.39	5920	18.10
3–5	2071	12.39	1881	11.76	3952	12.08
6–8	3708	22.19	3604	22.53	7312	22.35
9–11	2070	12.38	1929	12.06	3999	12.22
12–14	2816	16.85	2581	16.13	5397	16.50
15–18	2911	17.42	3221	20.13	6132	18.75
Overall	16,713	100	15,999	100	32,712	100
*Adults*^*a*^						
*Age (years)*						
18-	5394	11.20	4182	9.53	9576	10.41
30-	11,296	23.47	7858	17.91	19,154	20.82
40-	11,111	23.08	9784	22.31	20,895	22.71
50-	10,866	22.57	9604	21.90	20,470	22.25
60-	6064	12.60	8742	19.93	14,806	16.09
70-	3408	7.08	3695	8.42	7103	7.72
Overall	48,139	100	43,865	100	92,004	100
*Educational level*						
Illiterate	637	1.32	2070	4.72	2707	2.94
Elementary school	2474	5.14	4446	10.14	6920	7.52
Junior high school	10,528	21.87	8683	19.79	19,211	20.88
Senior high school	12,393	25.74	8520	19.42	20,913	22.73
College	18,270	37.95	15,464	35.25	33,734	36.67
Postgraduate	3250	6.75	3496	7.97	6746	7.33
*Marital status*						
Unmarried	4433	9.21	4195	9.56	8628	9.38
Married/cohabitating	36,701	76.24	35,460	80.84	72,161	78.43
Divorced/separated	608	1.26	1044	2.38	1652	1.80
Widowed	469	0.97	2067	4.71	2536	2.76
Ever-smoke	26,006	54.02	1943	4.43	27,949	30.38
Ever-drink	27,419	56.96	4159	9.48	31,578	34.32
*BMI categories*^*b*^						
Under weight	857	1.78	1763	4.02	2620	2.85
Normal weight	15,008	31.18	19,949	45.48	34,957	38.00
Overweight	20,095	41.74	14,175	32.32	34,270	37.25
Obesity	10,711	22.25	6336	14.44	17,047	18.53

^a^Not included pregnant women in the Early-life BTH cohort. ^b^Underweight was defined as BMI < 18.5 kg/m^2^, 18.5 ≤ BMI < 24 as normal weight, 24 ≤ BMI < 28 as overweight, and BMI ≥ 28 as obesity. *BMI* body mass index, kg/m^2^

Face-to-face questionnaire interview was conducted by trained staff including items on (1) demographic and socioeconomic characteristics; (2) health-related lifestyle factors, such as alcohol intake, smoking status (active smoking, passive smoking, E-cigarette use), dietary patterns, physical activity and sedentary behavior, pollutant exposure; (3) personal medical history and medication use of NCDs and cancers; and (4) family history of NCDs and cancers. For pregnant women, infants, adolescents and occupational workers, specific questionnaires and physical check-up items were designed. Considering that long questionnaire may be burdensome for participants, consensus has been reached among collaborators to keep the interview as brief as possible.

Physical examination included anthropometry of height, weight, and body composition, measurement of blood pressure, electrocardiogram, grip strength, bone mineral density. In the cohort from early pregnancy to adolescents, information legally required from regular health check-ups for pregnant women, newborns, infants, children, and adolescents were collected.

Fasting blood sample (at least 8 h) was drawn from each participant except for infants to establish DLCC’s biobank. 122,006 blood samples have been collected through the baseline survey, which provides invaluable resource for future genetic epidemiology research. For participants aged 6 and above, 6–10 ml of venous blood was collected and stored properly. For infants born after 42 days, dried blood spots were collected.

In Visit 2 of DLCC, as one of the study sites, Guangdong province, has been reported with relatively higher incidence of laryngeal squamous cell carcinoma [9]. We additionally collected oropharyngeal swab for HPV test, as previous studies revealed association between oropharyngeal HPV infection and laryngeal squamous cell carcinoma [10].

### Long term follow-up

DLCC study is designed with long-term follow-up, not limited by the current funding period. Low lost to follow-up rate is vital for the success of prospective cohort studies. Therefore, in the consideration of study sites selection, support from the local government, involvement willingness of primary health care settings, and the capacity of staff to carry out long-time follow up were key factors.

Active follow-up mechanism was predominantly used in DLCC. The targeted outcomes of study interest included death, newly onset NCDs, the growth and health trajectory of newborns, children and adolescents, pregnancy adverse outcomes, lifestyle risk factors changing patterns, etc. Follow-up by repeated measurements was conducted with telephone follow-up as a supplementary method. Overall, there were 92.33% participants in Visit 1 have been followed up successfully for at least once. For pregnant women and children, the frequency of follow-up was higher, combined with regular physical examinations required by current health administrative strategies. For population in Visit 2, we plan to conduct follow-up every two years by repeated measurements. Since the baseline survey is completed in 2021, the first-time follow-up has not yet been initiated. The information collection methods used in the baseline and follow-ups were summarized in Table 3.

**Table 3:** Methods used for baseline and follow-up data collection in DLCC

	Baseline survey				Follow-up			
	Questionnaire interview	Physical examination	Biochemical tests	Health records matching	Repeated measurements	Telephone interview	Health records matching	Surveillance data matching
Early-life BTH cohort (2017-)	√	√	√	√	√		√	
BTH-MEC cohort (2017-)	√	√	√	√	√	√	√	√
CHCN-BTH cohort (2017-)	√	√	√		√	√		
OCC cohort (2017-)	√	√	√		√			
CHB cohort (2021-)	√	√	√		NA	NA	NA	NA

During the COVID-19 epidemic, special strategies have been implemented to enhance and facilitate the cohort construction and avoid lost to follow-up. First, we designed a smartphone-based App to help enroll subjects. The function of physical examination appointment was available (maximum 200 people each day to ensure social distancing), by which the residents could know the real-time available number for a certain day’s examination on the App. Second, traditional ways to lower transmission risk were also used such as mask wearing, hand sanitizer’s usage, social distancing, well-ventilated environment, etc. Third, for individuals who were not able to participate in the field survey, telephone interviews were conducted after strict interviewer training.

### Data management and resource access

All data collected in DLCC were given unique identifiers. Various quality control strategies have been used to cover the whole process of DLCC. Especially, On-Site Quality Control has been emphasized. In the community-based cohorts, questionnaires were recovered immediately when individuals completed the physical examination. Experienced epidemiologists have been designated to re-check the completeness and correctness of each questionnaire before the participants leave the survey site. Project administrative meetings were held regularly to summarize, discuss, and develop potential coping strategies for emerging problems and concerns.

Under the consideration of project management and data sharing, the data of DLCC have been linked to the National Population Health Data Center (https://www.ncmi.cn/index.html). On which researchers can find descriptive information of the sub-cohorts included in DLCC, such as the introduction of datasets, methods used in collecting data, data quality control strategies, methods for statistical analyses, relevant publications, etc. Individual data access rights are assigned to institutes according to their role in the study.

### Key findings, productions, and social benefits

As DLCC is a newly established and still ongoing project, most publications were based on its baseline data, focusing on the health hazards of ambient air pollutants, associated factors of NCDs and health profiles in diverse populations (aged people, pregnant women, infants, or people with occupational exposures. For example, exposure of ambient air pollutants was found to be associated with cardiometabolic health and influence the process of inflammation in adults [7, 11, 12]. Associated factors with NCDs or cardiometabolic conditions were also explored [13–15]. Practical metabolic related diagnostic criterion for youths has been explored for better NCDs prevention initiated from childhood [16]. Machine learning and novel statistical analysis methods were applied in the NCDs risk prediction [17, 18]. Specific occupational exposures, such as rotating night shift and exposure to light at night, have been found to be associated with health disorders [19].

Several software for health assessment have been designed, such as the children and adolescents’ muscle fitness assessment system (Copyright certificate No. 2019SR0670482), hypertension and dyslipidemia assessment system for Chinese children and adolescents (Copyright certificate No. 2017SR646084 and 2019SR0670308). One patent on SNPs in identifying childhood obesity has been awarded by the National Patent Office (Certificate No. 3775678).

DLCC is a multidisciplinary project. By the end of 2021, more than 600 researchers have been involved in the project, covering the fields of public health and preventive medicine, clinical medicine, basic medicine, nursing, computer science and management science. Their effective collaboration brings organization framework that can be used by the support of a network of colleagues. In addition, in-depth free clinical consultation was carried out during the survey, leading by senior physicians from Top Hospitals in China. Trainings on physical examination, common chronic disease prevention and control were conducted for local healthcare providers. These activities provide the project considerable and sustainable social benefit.

### Strengths and limitations

DLCC has several unique and special features that make it a valuable resource for scientific research. First, the large sample size of the comparable prospective cohort study covers the whole life course of general population, allows diverse research areas, and provides a comprehensive database for further study. Under the management of consistent key team members, standardized questionnaire interview and measurement methodology were utilized in the process of data collection during Visit 1 and Visit 2, allowing for directly comparing individuals at different visits as well as in the follow-ups. Although some baseline characteristics among sub-cohorts are different, that is to be expected and we are developing an online platform for data standardization and sharing, which is available at (http://59.108.16.234:8082). Standardized data is required when uploading data to the platform, following certain rules on data coding rationale, dataset structure, health outcome definition and measurement, etc. Second, we used diverse information technology to improve the cohort establishment. For instants, to collect the subject’s basic information and identify follow-up status, a citizen identification card reader and a computer-based software specially designed for DLCC were used. The software also allows real-time transcription from hard copy of questionnaires into electronic format. Such technology, combined with the strict on-site-quality control strategy, guarantees the reliability of data, and provides models for other population-based studies. Third, the storage of blood samples enables assessment of genetic and other molecular factors as determinants or risk factors for various health outcomes in the future. In addition, in DLCC, most of the multi-health profiles were measured directly by face-to-face questionnaires, physical examinations, or laboratory biochemical tests. The repeated measurement during the follow-up could provide more accurate and objective data on capturing dynamics change of health conditions, providing valuable resource for health estimation.

The limitations of DLCC should also be acknowledged. Information on lifestyle and medical history collected by questionnaires may challenge the accuracy of data. However, effective training on questionnaire skills may reduce this bias to an acceptable level. Limited by the cohort-design nature, causal inference may be challenging based on the original data. Advanced methodology, such as mendelian randomization or other inference statistical methods would be used to yield better causal-inference exploration.

### Collaboration

DLCC study is conducted by multiple research groups that under integrated systematic program management. The collaborators are: Capital University, Nankai University, Tianjin Women’s and Children’s Health Center, North China University of Science and Technology, Hebei Provincial Center for Disease Control and Prevention, Beijing Physical Examination Center, Chaoyang District Center for Disease Control and Prevention, Hebei Medical University, Capital Institute of Pediatrics, Beijing Hepingli Hospital, Guangdong General Hospital and Hebei University. Since the data collection is still ongoing, the number of collaborating groups is expected to increase in the coming future.
